# Glucose Activates Vagal Control of Hyperglycemia and Inflammation in Fasted Mice

**DOI:** 10.1038/s41598-018-36298-z

**Published:** 2019-01-30

**Authors:** Biju Joseph, Guilherme Shimojo, Zhifeng Li, Maria del Rocio Thompson-Bonilla, Roshan Shah, Alexandre Kanashiro, Helio C. Salgado, Luis Ulloa

**Affiliations:** 1 Department of Surgery, Rutgers-New Jersey Medical School, Newark, NJ 07103 USA; 2 Hospital “October 1st”, ISSSTE”, 1669 National Polytechnic Institute Ave, Mexico City, Mexico; 3 Department of Physiology, Medical School - University of São Paulo, Ribeirão Preto, SP 14049-900 Brazil; 4 Center for Immunity and Inflammation, Rutgers-New Jersey Medical School, Newark, NJ 07103 USA

## Abstract

Sepsis is a leading cause of death in hospitalized patients. Many experimental treatments may have failed in clinical trials for sepsis, in part, because they focused on immune responses of healthy animals that did not mimic the metabolic settings of septic patients. Epidemiological studies show an association between metabolic and immune alterations and over 1/3 of septic patients are diabetic, but the mechanism linking these systems is unknown. Here, we report that metabolic fasting increased systemic inflammation and worsened survival in experimental sepsis. Feeding and administration of glucose in fasted mice activated the vagal tone without affecting blood pressure. Vagal stimulation attenuated hyperglycemia and serum TNF levels in sham but only hyperglycemia in splenectomized mice. Vagal stimulation induced the production of dopamine from the adrenal glands. Experimental diabetes increased hyperglycemia and systemic inflammation in experimental sepsis. Fenoldopam, a specific dopaminergic type-1 agonist, attenuated hyperglycemia and systemic inflammation in diabetic endotoxemic mice. These results indicate that glucose activates vagal control of hyperglycemia and inflammation in fasted septic mice via dopamine.

## Introduction

Sepsis represents a leading cause of death in the ICU of modern hospitals killing around 250,000 Americans per year^1–3^. There is no effective treatment by the FDA for severe sepsis and most present therapies are supportive. Modern antibiotics are more efficient in controlling infections, but sepsis still remains a leading cause of death in the ICU^1,4^. Severe sepsis is characterized by the overwhelming production of inflammatory cytokines that causes deleterious systemic inflammation and lethal multiple organ failure. During infection, pathogen- and damage-associated molecular pattern (PAMPs & DAMPs) molecules activate the innate immune system to produce inflammatory cytokines such as Tumor Necrosis Factor (TNF) and interleukins. These inflammatory cytokines are critical to fighting infections, but excessive production of these factors causes septic shock, cardiovascular collapse, organ damage, and lethal multiple organ failure^5^. This process is not exclusive to sepsis, and deleterious systemic inflammation is a major clinical challenge in critical care. Infection, ischemia/reperfusion, hemorrhage/resuscitation, shock, and trauma can also induce the excessive production of inflammatory factors that can cause multiple organ failure^6^. Current studies focus on the mechanisms inducing systemic inflammation and the design of novel therapeutic strategies to control systemic inflammation in infectious and inflammatory disorders.

Sepsis represents a major scientific challenge in modern medicine with over 100 unsuccessful clinical trials^7^. Multiple clinical trials with new generations of antibiotics controlled the infections but did not control systemic inflammation and organ damage. Other clinical trials inhibiting specific inflammatory factors such as TNF or IL1 may have failed, in part, because they focused on single cytokines, but sepsis is a complex process with multiple inflammatory factors contributing to multiple organ failure^8,9^. Although most experimental approaches still focus on the immune system, sepsis is a complex process with both immune and metabolic alterations that contribute to multiple organ failure^10–15^. The CDC estimated that around 70% of the septic patients had chronic disorders or received hospital attention during the month before the diagnosis of sepsis^16^. A common metabolic alteration in septic patients is diabetes, around 1/3 of septic patients have diabetes that causes hyperglycemia, which increases systemic inflammation, organ damage and 90-day mortality in septic patients^10–21^. Still, most studies focus on experimental models on sepsis performed on ‘healthy’ animals that do not mimic the metabolic alterations observed in clinical settings^8,21^. This association between metabolic and immune alterations is also confirmed by multiple epidemiological studies. Thus, recent efforts focus on identifying the physiological mechanisms connecting metabolic and immune alterations and their clinical implications in infectious and inflammatory disorders. Here, we analyzed how metabolic fasting affects the pathogenesis and neuronal regulation of the innate inflammatory responses to bacterial endotoxin.

## Results

### Metabolic fasting increased systemic inflammation and worsened survival in endotoxemia

**Figure 1:**
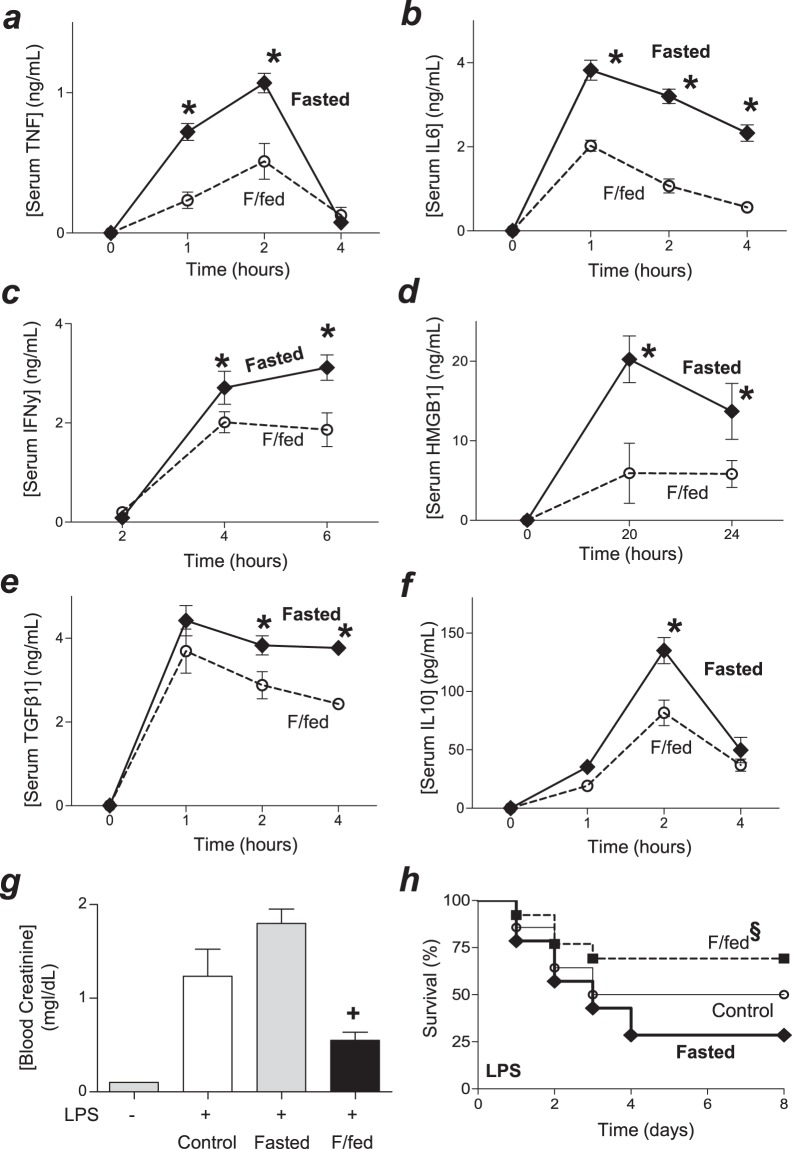
Fasting increases inflammation and mortality in endotoxemia. Mice were (control) fed ad libitum, (Fasted) fasted for 24 h, or (F/fed) fasted for 20 h and fed for 4 h before challenged with LPS (10 mg/kg; i.p.). Serum levels of TNF (**a**), IL6 (**b**), IFNγ (**c**), HMGB1 (**d**), TGFβ1 (**e**) and IL10 (**f**) were analyzed at the indicated time points. *p < 0.05 vs F/fed (n = 4/group, two-way ANOVA). (**g**) Blood creatinine levels at 24 h post-LPS. ^+^p < 0.05 vs F/fed (n = 4/group, one-way ANOVA). (**h**) Kaplan-Meier survival analyses of mice in endotoxemia (LPS 10 mg/kg; i.p.; ^§^p < 0.05 vs. F/fed, n = 20/group, Survival Log-rank test).

Epidemiological studies show an association between metabolic and immune alterations, but the mechanism linking these systems is unknown. Here, we analyzed whether metabolic fasting modulates the innate immune responses to bacterial endotoxin. First, we compared TNF production in control (fed ad libitum) and mice fasted for 24 hours. Fasted mice had slightly higher serum TNF levels but control mice had high variability of serum TNF levels that minimize the statistical significance (data not shown). We reasoned that this effect could be affected by postprandial metabolic markers and the variability in their feeding time. Thus, we added an experimental group of mice to synchronize their feeding time and postprandial metabolic markers after a fasted period. Mice were either (control) fed ad libitum, (Fasted) fasted for 24 hours, or (F/fed) fasted for 20 hours and fed for 4 hours before challenged with bacterial endotoxin (Fig. S1). The most significant differences were found in the higher serum TNF levels in fast as compared to the fasted/fed (F/fed) mice. We further analyzed these two experimental groups with time course analyses showing that fasted animals had higher serum levels of TNF and IL6 than fasted/fed animals (Fig. 1a,b). Fasted mice also had higher levels of ‘late’ inflammatory cytokines such as IFNγ and HMGB1, which are produced at 4–6 h and 18–24 h post-LPS, respectively (Fig. 1c,d). Fasted animals also had slightly higher levels of anti-inflammatory cytokines such as TGFβ1 and IL10 at 2 h post-LPS (Fig. 1e,f). Blood chemistry analyses did not show a significant difference between fasted and fasted/fed mice in blood levels of potassium, sodium, carbon dioxide, urea nitrogen or anion gap (Fig. S2a–f). However, fasted animals had higher creatinine blood levels at 24 h post-LPS and had significantly worse survival in endotoxemia than fasted/fed animals (Fig. 1g,h). Survival was recorded for two weeks and no late deaths were observed suggesting that fasting did not merely delay the pathology.

### Glucose activated the vagus nerve to attenuate TNF production

**Figure 2:**
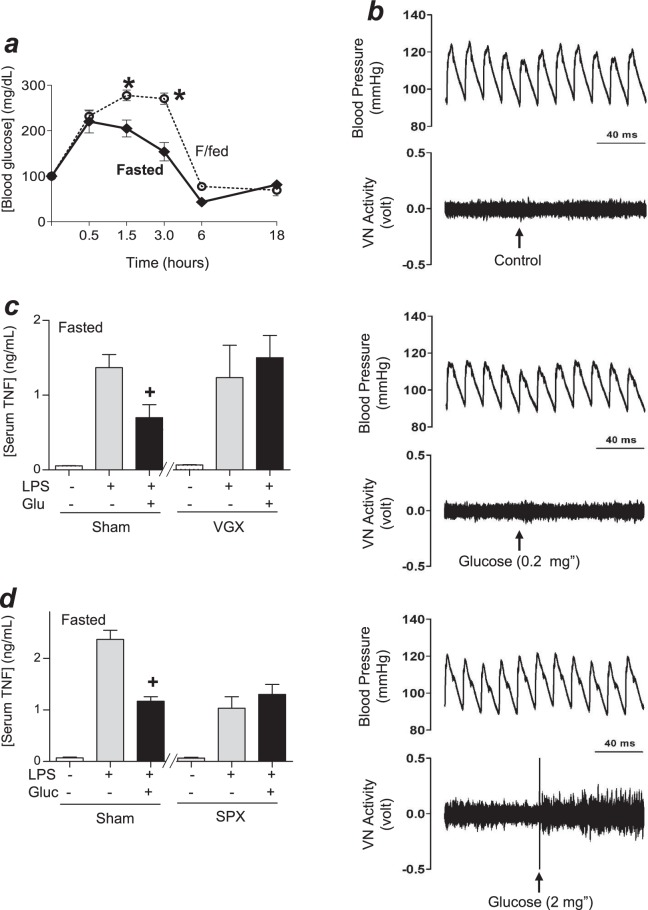
Glucose activates the vagus nerve. (**a**) Fasted or fasted/fed (F/fed) mice were challenged with LPS and blood glucose levels were analyzed at different time points. *p < 0.05 vs F/fed (n = 3/group, two-way ANOVA). (**b**) Fasted animals received saline solution with glucose(0, 0.2 or 2 mg; i.v.) and the vagal nerve activity and arterial blood pressure were recorded (n = 2-3/group for 3 sets of experiments performed for each of the treatments). (**c**,**d**) Fasted mice underwent sham or surgical (**c**) vagotomy (VGX), or (**d**) splenectomy (SPX) at 30 h or 3 days before LPS, respectively. Animals were treated with saline alone or with glucose (2 mg/Kg; i.p.), and serum TNF levels were analyzed at 1.5 h post-LPS. ^+^p < 0.05 vs LPS (n = 3/group; One-way ANOVA).

We next hypothesized that metabolic fasting may affect the hyperglycemic responses to bacterial infection. Fasted and fasted/fed mice had statistically similar blood glucose levels before endotoxemia, but the fasted/fed mice had higher hyperglycemic responses to endotoxemia (Fig. 2a). We also analyzed glucose during hypoglycemia at 18 h post-LPS, the latest time point when all mice still alive before mortality. This time point allowed us to analyze the highest number of mice and not the subpopulation of mice that survive the first onset of mortality, as 10–25% of mice died during the first 24 h after the LPS challenge. Our result didn’t show a significant difference in hypoglycemia between the experimental groups. Thus, we focused on the initial onset of hyperglycemia and whether it affects the production of inflammatory factors. Given that previous studies indicate that the vagal tone moderates the postprandial response to glycemic load^22^ and that the vagus nerve can inhibit TNF production in endotoxemia^23–25^, we reasoned that hyperglycemia may activate vagal modulation of TNF production in endotoxemia. Thus, we analyzed whether blood glucose activates the vagal tone. Intravenous acute administration of glucose did not affect arterial blood pressure but activated vagal electric potential difference in a concentration-dependent manner (Fig. 2b). Then, we analyzed whether this mechanism attenuates serum TNF levels. Administration of glucose decreased serum TNF levels through the vagus nerve, as it was prevented by surgical vagotomy (Fig. 2c). We previously reported that vagal electrical stimulation attenuates serum TNF levels in endotoxemic mice by inhibiting its production in the spleen^23–25^. Thus, we analyzed whether glucose induces a similar mechanism mediated by the spleen. Administration of glucose attenuated serum TNF levels in sham but not in splenectomized mice (Fig. 2d), similar to that described for vagal electrical stimulation^23–25^. These results indicate that glucose activates the vagus nerve to attenuate splenic and serum TNF levels in endotoxemia.

### Vagal stimulation attenuated hyperglycemia by inducing insulin

**Figure 3:**
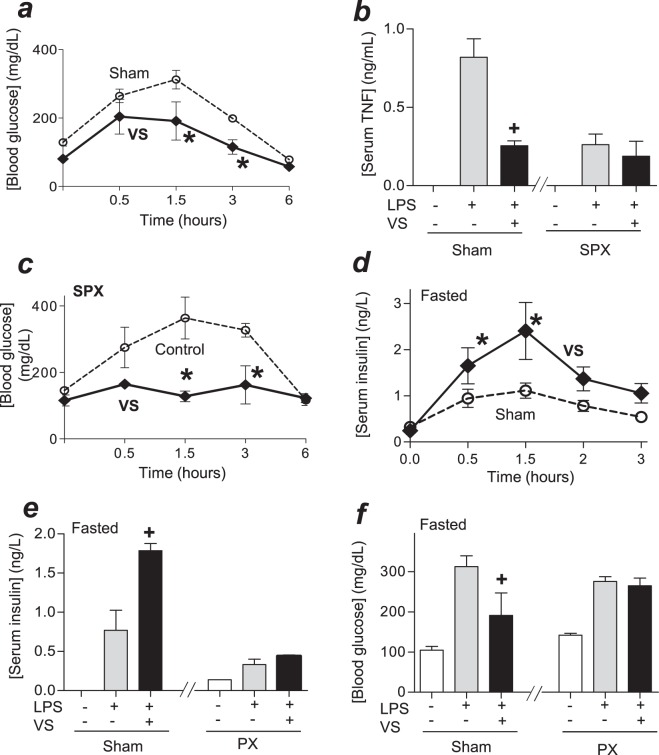
Vagal stimulation attenuates hyperglycemia by inducing insulin. (**a**) Mice were challenged with LPS and received sham or vagal stimulation (VS; 10 Hz, 60 min). Blood glucose levels were analyzed at different time points. *p < 0.05 vs sham (n = 4/group; Two-way ANOVA). (**b**,**c**) Mice underwent sham or surgical splenectomy (SPX) 3 days before LPS. Animals underwent control surgery or vagal stimulation (VS), and serum TNF at 1.5 h (**b**); ^**+**^p < 0.05 vs LPS (n = 3/group; One-way ANOVA), or blood glucose levels (**c**) were analyzed. *p < 0.05 vs LPS (n = 4/group; Two-way ANOVA). (**d**) Fasted mice received sham or vagal stimulation (VS), and serum insulin levels were analyzed at the indicated time points. *p < 0.05 vs LPS (n = 4/group; Two-way ANOVA). (**e**,**f**) Fasted mice underwent sham or surgical pancreatectomy (PX) 1 day before LPS, underwent sham or vagal stimulation, and blood insulin (**e**) or glucose (**f**) levels were analyzed at 1.5 h post-LPS. ^+^p < 0.05 vs LPS (n = 4/group; One-way ANOVA).

The previous studies on vagal modulation focused on TNF regulation during endotoxemia^23–25^. Here, we analyzed whether vagal stimulation attenuates hyperglycemia. Bacterial endotoxin induces hyperglycemia by over 3-fold and returned to normal levels after 3–4 h. Vagal electrical stimulation significantly attenuated hyperglycemic responses to bacterial endotoxin (Fig. 3a). Previous studies reported the potential of vagal stimulation to attenuate serum TNF levels^25–28^. We confirmed that vagal stimulation attenuates other inflammatory cytokines such as serum levels of IL-6 and IFNγ at 4 h post-LPS (Fig. S3). However, these cytokines are produced after the hyperglycemic peak, and thus we focused on the relationship between vagal modulation of hyperglycemic and TNF and whether the vagus nerve controls hyperglycemia by regulating TNF. As we previously reported that splenectomy abrogated vagal control of TNF, we analyzed whether splenectomy also prevents vagal modulation of hyperglycemia. Vagal electrical stimulation attenuated serum TNF levels in sham but not in splenectomized mice (Fig. 3b). By contrast, vagal electrical stimulation attenuated hyperglycemia in both sham and splenectomized mice (Fig. 3a,c). Next, we wondered whether the vagus nerve controls hyperglycemia by inducing insulin. Vagal stimulation increased serum insulin levels (Fig. 3d). We further confirmed our results in pancreatectomized mice. Vagal stimulation induced insulin in sham but not in pancreatectomized mice (Fig. 3e). Likewise, vagal stimulation attenuated hyperglycemia in sham but not in pancreatectomized mice (Fig. 3f). These results show that vagal stimulation can attenuate hyperglycemia in endotoxemia by inducing insulin.

### Dopaminergic control of hyperglycemia

**Figure 4:**
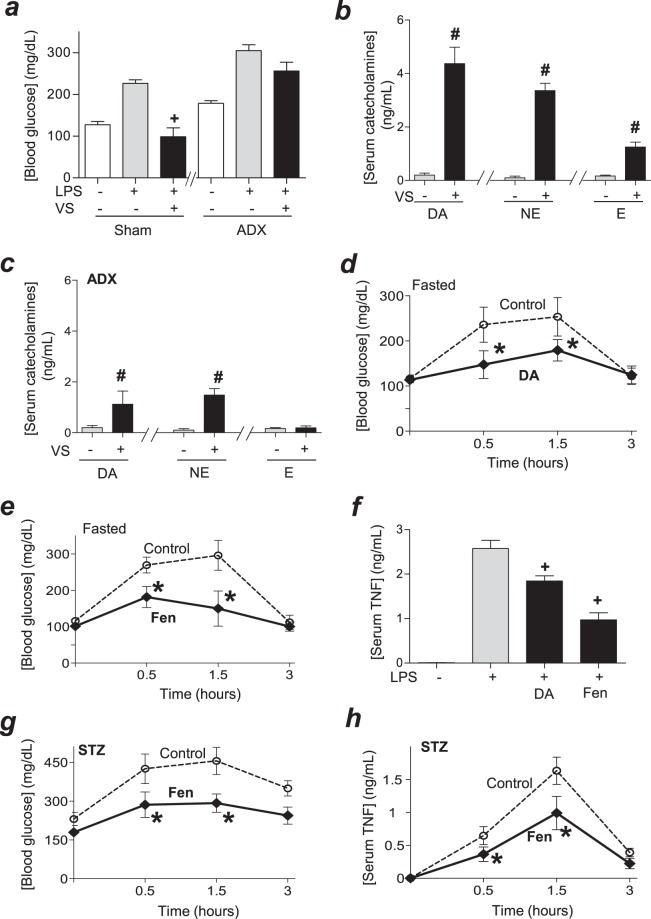
Dopamine controls hyperglycemia in experimental sepsis. (**a**–**c**) Fasted animals underwent (**a**) sham or (**a**,**c**) surgical adrenalectomy (ADX) 3 days before LPS. Then, animals underwent control or vagal stimulation (VS; 10 Hz, 15 min) right before the LPS challenge. (**a**) Blood glucose or (**b**,**c**) catecholamines (dopamine(DA), norepinephrine (NE) or epinephrine (E) were analyzed at 1.5 h post-LPS. (**d**–**f**) Fasted mice were treated with vehicle (control), dopamine (DA; 10 mg/kg/dose; i.p.) or fenoldopam (Fen; 10 mg/kg/dose; i.p.) at 6 and 1 h before the LPS challenge. (**d**,**e**) Blood glucose and (**f**) serum TNF levels were analyzed at the indicated time points. (**g**,**h**) All mice received streptozotocin (STZ; 40 mg/kg) and were treated with vehicle (control) or fenoldopam (Fen; 10 mg/kg/dose; i.p.) at 6 and 1 h before LPS. Blood glucose (**g**) and TNF (**h**) were analyzed at the indicated time points. (**a**,**f**) ^+^p < 0.05 vs LPS (n = 4/group; One-way ANOVA); (**b**,**c**) ^#^p < 0.05 vs LPS (n = 3/group; Student’s t-test); (**d**,**e**,**g**,**h**) *p < 0.05 vs control (n = 4/group, Two-way ANOVA).

Next, we analyzed how the vagus nerve induces insulin. Previous studies showed that the pancreas is innervated by ‘sensory’ vagal fibers that may regulate insulin production through a sensory/efferent splanchnic network that involve other organs^29–34^. These results concur with other studies showing that innervations of the adrenal glands modulate hyperglycemia in hemorrhagic shock^35,36^. Thus, we analyzed whether vagal stimulation attenuates hyperglycemia in adrenalectomized endotoxemic mice. Vagal stimulation attenuated hyperglycemia by around 2-fold in sham but not in adrenalectomized mice (Fig. 4a). Next, we analyzed whether vagal stimulation induces the production of catecholamines in the adrenal glands. Vagal stimulation induced the production of the three catecholamines: dopamine, norepinephrine, and epinephrine, but the most significant effect was the increase in blood dopamine levels (Fig. 4b). Conversely, the most significant effect of adrenalectomy was to attenuate the vagal induction of dopamine (Fig. 4c). Thus, we analyzed the potential of dopamine to attenuate hyperglycemia in endotoxemia. Treatment with dopamine attenuated hyperglycemia in endotoxemic mice (Fig. 4d) similar to that described with vagal stimulation. Given that dopamine has a short chemical half-life span limiting its clinical potential, we analyzed whether fenoldopam, a well-known stable specific agonist for D1-like dopaminergic receptors^37,38^, mimics the potential of dopamine to attenuate hyperglycemia. Fenoldopam was more effective than dopamine at inhibiting hyperglycemia in endotoxemia (Fig. 4e). Fenoldopam was also more effective than dopamine at attenuating serum TNF levels (Fig. 4f). These results show the potential of dopaminergic agonists to modulate both hyperglycemic and inflammatory responses to bacterial endotoxin.

We realized that most experimental models of sepsis are based on ‘healthy’ mice with normal immune and metabolic systems that do not represent the common comorbidity of septic patients. Among them, around 1/3 of septic patients have diabetes that causes hyperglycemia, which increases systemic inflammation, organ damage and 90-day mortality in septic patients^10–15,21^. We previously reported that the induction of diabetes with streptozotocin, the standard method to induce experimental diabetes in mice, increases hyperglycemic and inflammatory responses to bacterial endotoxin and worsens survival in endotoxemic mice^39^. Thus, we analyzed whether fenoldopam attenuates hyperglycemia in experimental sepsis with diabetes. Time-course experiments indicated that treatment with fenoldopam significantly attenuates hyperglycemia in endotoxemic diabetic mice from 0.5 to 3 hours post-LPS (Fig. 4g). The analyses of TNF in the same samples show that treatment with fenoldopam also attenuates serum TNF levels for over 3 hours after the LPS challenge (Fig. 4h). These time-course analyses show that fenoldopam inhibited and did not merely delay hyperglycemia or TNF production in endotoxemic mice with diabetes. These results suggest that dopaminergic agonist type 1, such a fenoldopam may attenuate hyperglycemia and inflammation in both normal and diabetic endotoxemic mice.

## Discussion

Our results indicate that metabolic fasting affects both hyperglycemic and systemic inflammatory responses to bacterial endotoxin by modulating vagal neuromodulation. Fasted animals had worse systemic inflammation, organ function, and survival in experimental sepsis. Thus, we analyzed the relationship between glycemia and the inflammatory response to bacterial endotoxin. Experimental and clinical studies have shown that sepsis is characterized by an initial hyperglycemic response followed by a hypoglycemic phase and both phases contribute to the pathogenesis and prognosis of sepsis^12–15,40^. Fasted/fed mice had a higher hyperglycemic spike in endotoxemia, but similar hypoglycemic levels than fasted animals. Thus, we focused on the initial hyperglycemic response and whether they determine the production of inflammatory factors. Indeed, administration of glucose activated vagal electric potential and attenuated serum TNF levels in sham but not in vagotomized mice. Although the molecular mechanisms of vagal activation by hyperglycemia are unknown, recent studies show that glucose activates specific neuronal networks in central regions that modulate vagal activity. Glucose can activate pro-opiomelanocortin (POMC), melanin-concentrating hormone (MCH) and neuropeptide Y(NPY)/agouti-related peptide (AgRP) neurons in the hypothalamic arcuate nucleus and the nucleus tractus solitarius^41–46^. These neurons can activate different neuronal networks with specific implications. For instance, defective autophagy in POMC neurons leads to glucose intolerance and obesity^47,48^, whereas disrupted autophagy in glucose-sensing AgRP neurons promotes leanness and reduces food intake^49,50^. Although recent studies suggested that glucose transporters and hexokinase can contribute to neuronal activation by glucose, the molecular mechanisms are not well-known^51–53^. Furthermore, glucose can also activate astrocytes, which in turn can activate central neuronal networks through the astrocyte-neuron lactate shuttle^46^ and the neuronal pyruvate metabolism^54^. These studies focused on the potential of glucose to activate central neuronal networks that regulate energy homeostasis and hyperglycemia. Our results now warrant similar studies to determine the specific neurons and networks activated by glucose to induce vagal control of hyperglycemia and inflammation in infectious disorders. Future studies will also determine how these networks may link metabolic and immunological disorders and their contribution to inflammatory or infectious disorders such as sepsis.

Administration of glucose activated vagal electrical potential without affecting arterial blood pressure. These effects are similar to that observed with intracerebroventricular injection of semapimod, a drug currently used in clinical trials for Crohn’s disease^55^. Semapimod also activated vagal electrical potential and attenuated serum TNF levels in endotoxemia without affecting arterial blood pressure^55^. Likewise, melanocortin peptides, cholecystokinin, ghrelin, and leptin can also activate the vagus nerve to control physiological homeostasis^55–57^. These mechanisms of vagal activation have multiple clinical implications. For instance, deletion of leptin-induced vagal activation causes hyperphagia, obesity, diabetes, and infertility^56,57^. Likewise, multiple laboratories have shown that vagal stimulation attenuated TNF production in arthritis^58^, ischemia/reperfusion^59–61^, hemorrhage/resuscitation^61^, pancreatitis^62^, endotoxemia^25,26^ and severe sepsis^63,64^. Although these studies focused on vagal regulation of serum TNF levels, our present study now suggests that vagal regulation of hyperglycemia can also contribute to the effects of the vagus nerve in these conditions. In line with the hypothesis, our recent clinical study shows that neuronal stimulation can improve postoperative recovery by preventing hyperglycemia^65^.

Our results indicate that efferent vagal stimulation attenuates hyperglycemia in endotoxemia by inducing insulin and regardless of TNF regulation. Although neuronal regulation of insulin secretion is a complex process depending on the physiological conditions, our results concur with previous studies indicating that the vagus nerve can modulate cephalic and postprandial insulin secretion^29–31^. From a clinical perspective, patients with complete resection of the subdiaphragmatic vagus nerve can display a longer periodicity of plasma insulin oscillations^32^. Clinical studies on patients with subdiaphragmatic vagotomy also indicate that truncal vagotomy decreases the glucagon response to insulin hypoglycemia as compared to selective vagotomy^66^, and abdominal vagal blocking decreases pancreatic exocrine secretion in the animal model^67^. Furthermore, recent studies indicate that vagal blocking can improve glycemic control and blood pressure in obese patients with type 2 diabetes mellitus^68^. Previous studies also showed that the pancreas is innervated by ‘sensory’ vagal fibers from the hepatic subdiaphragmatic branches and suggest that the vagus nerve may regulate insulin production through a complex sensory/efferent splanchnic network that involves other organs^29–34^. In agreement with these results, previous physiological studies showed that innervations of the adrenal glands modulate hyperglycemia in hemorrhagic shock^35,36^. Our current results show that efferent vagal stimulation induced insulin and adrenalectomy prevented vagal control of hyperglycemia in endotoxemia.

Our study shows that vagal stimulation induces the production of dopamine from the adrenal glands. Administration of dopamine decreased hyperglycemia and serum TNF levels in endotoxemia. These results concur with clinical studies showing that dopamine can restore tissue perfusion in septic shock and critically ill patients^69,70^. However, dopamine can also increase the risk of tachyarrhythmia^69,70^ and worsens survival in septic animals^71^. We hypothesized that selective dopaminergic agonists may avoid unspecific side effects of dopamine. Dopaminergic receptors are classified into D1-like (D1R, D5R) or D2-like (D2R, D3R, D4R) receptors that signal through Gαs or Gαi proteins, respectively. Fenoldopam is a well-characterized, stable and specific agonist with ~100-fold greater affinity for D1Rs than dopamine^35–38^. Fenoldopam was more effective than dopamine at controlling hyperglycemia and TNF production in endotoxemic mice. However, most experimental models of sepsis are based on ‘healthy’ mice with normal immune and metabolic systems that do not resemble the common preexisting conditions of septic patients^10–15^. Indeed, over 1/3 of septic patients have diabetes that causes hyperglycemia, which increases systemic inflammation, organ damage and 90-day mortality in septic patients^10–15,21^. We previously reported that experimental diabetes increases hyperglycemic and inflammatory responses to bacterial endotoxin and worsens survival in endotoxemic mice^39^. Our present study indicates that treatment with fenoldopam attenuates, and doesn’t merely delay, both hyperglycemia and serum TNF levels for over 3 hours after the LPS in endotoxemic mice with diabetes. Our present results also suggest that fenoldopam attenuates hyperglycemia before the serum TNF peak at 1.5 h post-LPS, and thus the effects of fenoldopam on glucose may precede its effects on serum TNF levels. Future studies will be required to determine whether fenoldopam control of hyperglycemia contributes to modulate serum TNF levels. These results concur with our previous studies showing that treatment with fenoldopam, started at 15 h after the cecal ligation and puncture, improves survival in diabetic mice with established polymicrobial peritonitis induced by cecal ligation and puncture^39^. By comparison, inhibition of TNF by using neutralizing anti-TNF antibodies increased the mortality when administered after the septic challenge in normal mice^9^. These studies of neuromodulation are allowing the design of novel treatments for infectious and inflammatory disorders. The study of the sympathetic baroreflex system allowed the design of selective beta-blockers for hypertension and arrhythmia. Similar studies are now required to determine whether specific dopaminergic agonists may provide therapeutic advantages for hyperglycemia and inflammation in septic patients with preexisting metabolic conditions such as diabetes.

## Material and Methods

### Chemicals and Reagents

LPS (*E*. *Coli* 0111: B4), dopamine hydrochloride, fenoldopam, streptozotocin, and glucose were purchased from Sigma-Aldrich® (Saint Louis, MO). The glucose measuring strips were purchased from PharmaTech Solutions, Inc (Westlake Village, CA). Pentobarbital sodium was purchased from Diamondback (Scottsdale, AZ); ketamine from Henry Schein animal health (Dublin, OH); xylazine from Akron animal health (Lake Forest, IL, USA) and enrofloxacin from Bayer Healthcare (Shawnee Mission, KS, USA). Dopamine (DA; 10 mg/kg/dose; i.p.) or fenoldopam (Fen; 10 mg/kg/dose; i.p.) were administered at 6 and 1 h before LPS.

### Animal Experiments

All experimental procedures were approved by the Institutional Animal Care & Use Committee of the Rutgers New Jersey Medical School and adhered to *The Guide for the Care and Use of Laboratory Animals* by the National Academy of Sciences as published by the National Institutes of Health (Copyright© 1996 by the National Academy of Sciences). Briefly, C57BL/6J 6–8 week old male mice were obtained from Charles River Laboratories (Wilmington, MA). Mice were fed standard chow diet (24.7% energy from protein, 62.1% energy from carbohydrate, 13.2% energy from fat; PicoLab Rodent Diet 20 from Labdiet, St. Louis, MO). The investigators analyzing the samples were blinded to the treatments. *Endotoxemia*: Endotoxin (10 mg/kg, i.p., *E*. *coli* LPS 0111:B4) was dissolved in sterile, pyrogen-free PBS, and sonicated for 20 mins. *Diabetes*: Streptozotocin was injected (STZ; 40 mg/kg; i.p.) 10 days before the experiment as reported^72–74^.

### Vagotomy and Electrical Stimulations

Animals were anesthetized with pentobarbital (50 mg/kg, i.p.) and vagal surgeries performed as we described^38^. Surgical Cervical Vagotomy (VGX) A ventral incision on the neck was performed to retract the sternocleidomastoid muscle and to visualize the carotid artery and vagus nerves. The vagal trunks were ligated with size 4–0 silk and sectioned. *Vagal Stimulation* (VS) The right cervical vagal trunk was isolated and connected to the platinum electrode. Electrical stimulation was performed for 60 mins at 5 V, with 40 mA, a pulse width of 200 μs, and a frequency of 10 Hz using the electrostimulator (STM 150, Biopac Systems, Goleta, CA). Control animals underwent sham surgery without the electrical stimulation. *Vagal activity recording* was performed as described^75^. Anesthetized Wistar rats received ventral neck incision to connect the vagus nerve to the electrode. Then, the carotid artery and femoral vein were catheterized for recording pulsatile arterial pressure and for glucose administration (iv), respectively. Animals were allowed to recover consciousness after surgery. The nerve activity was recorded with the impedance pre-amplifier Princeton Applied Research-113 and filtered 3–100 kHz. The arterial catheter was connected to a pressure transducer MLT844 and the signal was amplified by ML224 (ADInstruments, Bella Vista, Australia) and sampled in the oscilloscope Tektronics-5113.

### Ablative Surgeries

Animals were anesthetized with 100 mg/kg ketamine; 20 mg/kg xylazine, i.p. Anesthesia was confirmed by the absence of withdrawal reflex to toe pinch. Antibiotics (Enrofloxacine 2.5 mg/kg, s.c.; Baytril®, Bayer Health Care™, Swanee Mission, KA) were given to those animals with ablative surgery started right after surgery and given every 12 h until 24 h before the endotoxic challenge. *Splenectomy:* was performed 3 days prior to the experiment as we described in J Exp Med^25^. Animals underwent an abdominal incision to expose the spleen. The three main branches of the splenic artery were stabilized with nylon thread, ligated and cut. The spleen was removed and the wound was closed with sutures with catgut for the abdominal wall, and nylon thread for the skin. *Adrenalectomy:* was performed as described^76^. Animals received a dorsal incision from the first to the third lumbar vertebrae. The latissimus dorsi muscle was retracted and both adrenal glands and their adipose tissue were removed. *Pancreatectomy*: was performed similarly to that previously described^77^. Briefly, animals underwent a ventral left lateral abdominal incision to isolate and remove the pancreas 24 h before the LPS challenge.

### Blood and Serum Analyses

Blood was collected from a cardiac puncture at the indicated time points, allowed to clot for 30 mins at room temperature, and centrifuged at 2,000 g for 5 minutes at 4 °C similar as previously described^38^. TNF, IL6, IL10, TGFβ1 and IFNγ were analyzed with the respective ELISA kit (Affymettrix Inc, San Diego, CA). Serum HMGB1 was analyzed using HMGB1 detection ELISA kit (Chondrex Inc., Redmond, WA). Blood catecholamines were analyzed by ELISA (LDN immunoassays and services, Germany) at 1.5 h post-stimulation as we described^23,76^. Glucose was analyzed from the mouse tail tip blood using the Genstrip (PharmaTech Solutions Inc., Westlake Village, CA) and the OneTouch UltraMini glucometer, (LifeScan Inc., Milpitas, CA). Serum insulin was analyzed with the Mercodia mouse insulin ELISA kit, (Mercodia AB, Uppsala, Sweden). Blood chemistry analyses were performed at 24 h post-LPS with the i-Stat blood analyzer (Abbot Laboratories, Ilinois).

### Statistical Analyses

Tests were performed with GraphPad Prism Software® (GraphPad Software, La Jolla, CA). The sample size was determined with power analyses of our previous studies^25,76^. The figures are representative of experiments that were repeated twice in different days with n = 3–4 per experiment, and the data are expressed as mean ± standard error (SEM). The Student’s t-test (Mann–Whitney U test) was used to compare two experimental groups. Analyses of three or more groups were performed with one-way ANOVA with multiple pair-wise comparisons. The time courses and pair-wise comparisons were analyzed with the two-way ANOVA for repeated measures. Pair comparisons in ANOVA nonparametric tests were *post-hoc* adjusted with Tukey test (in equal sample sizes) or Bonferroni’s for multiple hypothesis testing. Normality and homogeneity of variance were confirmed with Kolmogorov-Smirnov analyses. Survival was analyzed with the Log-rank (Mantel-Cox) test. p < 0.05 are considered statistically significant and represented: ^#^Student’s t-test, ^+^One-way ANOVA, *Two-way ANOVA, ^§^Survival Log-rank test.

## Electronic supplementary material

Supplementary Figures

## Data Availability

The authors will make materials, data and associated protocols promptly available to readers.

## References

[1] Angus DC (2001). Epidemiology of severe sepsis in the United States: analysis of incidence, outcome, and associated costs of care. Critical care medicine.

[2] Hotchkiss RS, Karl IE (2003). The pathophysiology and treatment of sepsis. The New England journal of medicine.

[3] Ulloa L, Tracey KJ (2005). The ‘cytokine profile’: a code for sepsis. Trends Mol Med.

[4] Rice TW, Bernard GR (2005). Therapeutic intervention and targets for sepsis. Annu Rev Med.

[5] Martin GS, Mannino DM, Eaton S, Moss M (2003). The epidemiology of sepsis in the United States from 1979 through 2000. The New England journal of medicine.

[6] Tracey KJ (1987). Anti-cachectin/TNF monoclonal antibodies prevent septic shock during lethal bacteraemia. Nature.

[7] Marshall JC (2014). Why have clinical trials in sepsis failed?. Trends Mol Med.

[8] Carré JE, Singer M (2008). Cellular energetic metabolism in sepsis: The need for a systems approach. Biochimica et Biophysica Acta (BBA) - Bioenergetics.

[9] Eskandari MK (1992). Anti-tumor necrosis factor antibody therapy fails to prevent lethality after cecal ligation and puncture or endotoxemia. J Immunol.

[10] Brealey D (2002). Association between mitochondrial dysfunction and severity and outcome of septic shock. Lancet (London, England).

[11] Wolfe RR, Shaw JH, Durkot MJ (1983). Energy metabolism in trauma and sepsis: the role of fat. Progress in clinical and biological research.

[12] Machado FR (2017). The epidemiology of sepsis in Brazilian intensive care units (the Sepsis PREvalence Assessment Database, SPREAD): an observational study. The Lancet Infectious Diseases.

[13] Cerra FB (1989). Hypermetabolism-organ failure syndrome: a metabolic response to injury. Critical care clinics.

[14] van Vught LA, Holman R, de Jonge E, de Keizer NF, van der Poll T (2017). Diabetes Is Not Associated With Increased 90-Day Mortality Risk in Critically Ill Patients With Sepsis. Critical care medicine.

[15] Finfer S (2009). Intensive versus conventional glucose control in critically ill patients. The New England journal of medicine.

[16] Novosad SA (2016). Vital Signs: Epidemiology of Sepsis: Prevalence of Health Care Factors and Opportunities for Prevention. MMWR Morb Mortal Wkly Rep.

[17] Kosteli A (2010). Weight loss and lipolysis promote a dynamic immune response in murine adipose tissue. The Journal of Clinical Investigation.

[18] Weisberg SP (2006). CCR2 modulates inflammatory and metabolic effects of high-fat feeding. J Clin Invest.

[19] Arkan MC (2005). IKK-beta links inflammation to obesity-induced insulin resistance. Nat Med.

[20] Solinas G (2007). JNK1 in hematopoietically derived cells contributes to diet-induced inflammation and insulin resistance without affecting obesity. Cell metabolism.

[21] Buras JA, Holzmann B, Sitkovsky M (2005). Animal models of sepsis: setting the stage. Nat Rev Drug Discov.

[22] Young HA, Watkins H (2016). Eating disinhibition and vagal tone moderate the postprandial response to glycemic load: a randomised controlled trial. Scientific reports.

[23] Vida G (2011). β2-Adrenoreceptors of regulatory lymphocytes are essential for vagal neuromodulation of the innate immune system. FASEB J.

[24] Wang H (2003). Nicotinic acetylcholine receptor alpha 7 subunit is an essential regulator of inflammation. Nature.

[25] Huston JM (2006). Splenectomy inactivates the cholinergic antiinflammatory pathway during lethal endotoxemia and polymicrobial sepsis. J Exp Med.

[26] Borovikova LV (2000). Vagus nerve stimulation attenuates the systemic inflammatory response to endotoxin. Nature.

[27] Ulloa L, Quiroz-Gonzalez S, Torres-Rosas R (2017). Nerve Stimulation: Immunomodulation and Control of Inflammation. Trends Mol Med.

[28] Kanashiro A, Bassi GS, Cunha FQ, Ulloa L (2018). From Neuroimunomodulation To Bioelectronic Treatment Of RheumatoidArthritis. Bioelectronic Med.

[29] Ahren B, Holst JJ (2001). The cephalic insulin response to meal ingestion in humans is dependent on both cholinergic and noncholinergic mechanisms and is important for postprandial glycemia. Diabetes.

[30] Li Y, Hao Y, Owyang C (1997). High-affinity CCK-A receptors on the vagus nerve mediate CCK-stimulated pancreatic secretion in rats. The American journal of physiology.

[31] Niijima A (1981). Visceral afferents and metabolic function. Diabetologia.

[32] Travagli RA, Anselmi L (2016). Vagal neurocircuitry and its influence on gastric motility. Nature reviews. Gastroenterology & hepatology.

[33] Niijima A (1983). Glucose-sensitive afferent nerve fibers in the liver and their role in food intake and blood glucose regulation. J Auton Nerv Syst.

[34] Yamaguchi N (1992). Sympathoadrenal system in neuroendocrine control of glucose: mechanisms involved in the liver, pancreas, and adrenal gland under hemorrhagic and hypoglycemic stress. Can J Physiol Pharmacol.

[35] Tiberi M, Caron MG (1994). High agonist-independent activity is a distinguishing feature of the dopamine D1B receptor subtype. The Journal of biological chemistry.

[36] Balbo SL (2016). Vagotomy diminishes obesity in cafeteria rats by decreasing cholinergic potentiation of insulin release. J Physiol Biochem.

[37] Grenader A, Healy DP (1991). Fenoldopam is a partial agonist at dopamine-1 (DA1) receptors in LLC-PK1 cells. J Pharmacol Exp Ther.

[38] Torres-Rosas R (2014). Dopamine mediates vagal modulation of the immune system by electroacupuncture. Nat Med.

[39] Feketeova E (2018). Dopaminergic Control of Inflammation and Glycemia in Sepsis and Diabetes. Front Immunol.

[40] Weis S (2017). Metabolic Adaptation Establishes Disease Tolerance to Sepsis. Cell.

[41] Kong D (2010). Glucose stimulation of hypothalamic MCH neurons involves K(ATP) channels, is modulated by UCP2, and regulates peripheral glucose homeostasis. Cell metabolism.

[42] Grill HJ, Hayes MR (2012). Hindbrain neurons as an essential hub in the neuroanatomically distributed control of energy balance. Cell metabolism.

[43] Mergenthaler P, Lindauer U, Dienel GA, Meisel A (2013). Sugar for the brain: the role of glucose in physiological and pathological brain function. Trends in neurosciences.

[44] Ruud J, Steculorum SM, Brüning JC (2017). Neuronal control of peripheral insulin sensitivity and glucose metabolism. Nature Communications.

[45] Udit S (2017). Nav1.8 neurons are involved in limiting acute phase responses to dietary fat. Mol Metab.

[46] Lam CK, Chari M, Lam TK (2009). CNS regulation of glucose homeostasis. Physiology (Bethesda).

[47] Coupe B (2012). Loss of autophagy in pro-opiomelanocortin neurons perturbs axon growth and causes metabolic dysregulation. Cell metabolism.

[48] Kaushik S (2012). Loss of autophagy in hypothalamic POMC neurons impairs lipolysis. EMBO Rep.

[49] Kaushik S (2011). Autophagy in hypothalamic AgRP neurons regulates food intake and energy balance. Cell metabolism.

[50] Kroemer G, Marino G, Levine B (2010). Autophagy and the integrated stress response. Mol Cell.

[51] Jordan SD, Konner AC, Bruning JC (2010). Sensing the fuels: glucose and lipid signaling in the CNS controlling energy homeostasis. Cell Mol Life Sci.

[52] Levin BE (2006). Metabolic sensing neurons and the control of energy homeostasis. Physiol Behav.

[53] Marty N, Dallaporta M, Thorens B (2007). Brain glucose sensing, counterregulation, and energy homeostasis. Physiology (Bethesda).

[54] Lam TKT, Gutierrez-Juarez R, Pocai A, Rossetti L (2005). Regulation of Blood Glucose by Hypothalamic Pyruvate Metabolism. Science.

[55] Bernik TR (2002). Pharmacological stimulation of the cholinergic antiinflammatory pathway. J Exp Med.

[56] Gao Q (2004). Disruption of neural signal transducer and activator of transcription 3 causes obesity, diabetes, infertility, and thermal dysregulation. Proc Natl Acad Sci USA.

[57] de Lartigue G, Ronveaux CC, Raybould HE (2014). Deletion of leptin signaling in vagal afferent neurons results in hyperphagia and obesity. Mol Metab.

[58] Koopman FA (2016). Vagus nerve stimulation inhibits cytokine production and attenuates disease severity in rheumatoid arthritis. Proc Natl Acad Sci USA.

[59] Bernik TR (2002). Cholinergic antiinflammatory pathway inhibition of tumor necrosis factor during ischemia reperfusion. J Vasc Surg.

[60] Altavilla D (2006). Activation of the cholinergic anti-inflammatory pathway reduces NF-kappab activation, blunts TNF-alpha production, and protects againts splanchic artery occlusion shock. Shock (Augusta, Ga.).

[61] Cai B (2009). Alpha7 cholinergic-agonist prevents systemic inflammation and improves survival during resuscitation. J Cell Mol Med.

[62] Van Westerloo DJ (2006). The vagus nerve and nicotinic receptors modulate experimental pancreatitis severity in mice. Gastroenterology.

[63] Wang H (2004). Cholinergic agonists inhibit HMGB1 release and improve survival in experimental sepsis. Nat Med.

[64] van Westerloo DJ (2005). The cholinergic anti-inflammatory pathway regulates the host response during septic peritonitis. J Infect Dis.

[65] Grech D (2016). Intraoperative Low-frequency Electroacupuncture under General Anesthesia Improves Postoperative Recovery in a RandomizedTrial. J Acupunct Meridian.

[66] Bloom SR, Vaughan NJ, Russell RC (1974). Vagal control of glucagon release in man. Lancet (London, England).

[67] Tweden KS (2006). Vagal blocking for obesity control (VBLOC): studies of pancreatic and gastric function and safety in a porcine model. Surgery For Obesity and Related Diseases.

[68] Shikora S (2013). Vagal blocking improves glycemic control and elevated blood pressure in obese subjects with type 2 diabetes mellitus. Journal of obesity.

[69] Povoa PR, Carneiro AH, Ribeiro OS, Pereira AC (2009). Influence of vasopressor agent in septic shock mortality. Results from the Portuguese Community-Acquired Sepsis Study (SACiUCI study). Critical care medicine.

[70] Sakr Y (2006). Does dopamine administration in shock influence outcome? Results of the Sepsis Occurrence in Acutely Ill Patients (SOAP) Study. Critical care medicine.

[71] Oberbeck R (2006). Dopamine affects cellular immune functions during polymicrobial sepsis. Intensive care medicine.

[72] Graham ML, Janecek JL, Kittredge JA, Hering BJ, Schuurman H-J (2011). The Streptozotocin-Induced Diabetic Nude Mouse Model: Differences between Animals from Different Sources. Comparative Medicine.

[73] Deeds MC (2011). Single Dose Streptozotocin Induced Diabetes: Considerations for Study Design in Islet Transplantation Models. Laboratory animals.

[74] Wu KK, Huan Y (2008). Streptozotocin-induced diabetic models in mice and rats. Curr Protoc Pharmacol.

[75] Licursi de Alcantara AC, Salgado HC, Sassoli Fazan VP (2008). Morphology and morphometry of the vagus nerve in male and female spontaneously hypertensive rats. Brain research.

[76] Vida G, Peña G, Deitch EA, Ulloa L (2011). Alpha7-nicotinic receptor mediates vagal induction of splenic norepinephrine. J Immunol.

[77] Davies JE, White SA, Clayton HA, Swift SM, Dennison AR (1998). Inflammatory response after total pancreatectomy and islet autotransplantation. Transplantation proceedings.

