# Supplementary material for: Glucose Activates Vagal Control of Hyperglycemia and Inflammation in Fasted Mice
Source: Sci Rep. 2019 Jan 30;9:1012. doi: 10.1038/s41598-018-36298-z (PMC6354016; doi:10.1038/s41598-018-36298-z)
Supplement: Supplementary file 1 — Supplementary Figures [file 41598_2018_36298_MOESM1_ESM.pdf]

Glucose Activates Vagal Control Of hyperglycemia And Inflammation In Fasted Mice.

Biju Joseph, Guilherme Shimojo, Zhifeng Li, Maria del Rocio Thompson-Bonilla, Roshan Shah, Alexandre Kanashiro, Helio C. Salgado & Luis Ulloa.

Supplementary Figures.

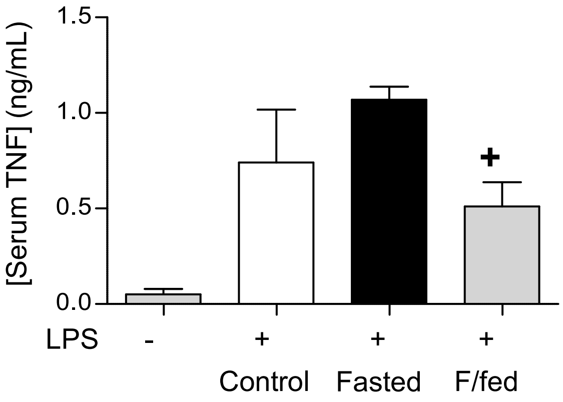

<u>Supplementary Fig.1.</u> **<u>Serum TNF levels in control and fasted mice in endotoxemia.</u>**
(a) Mice were (control) fed ad libitum, (fasted) fasted for 24h, or (F/fed) fasted for 20 and fed for 4h before the LPS challenge(10 mg/kg; i.p.). Serum TNF levels were analyzed at 1.5h post-LPS. +$p<0.05$ vs fasted (n=4/group, One-way ANOVA).

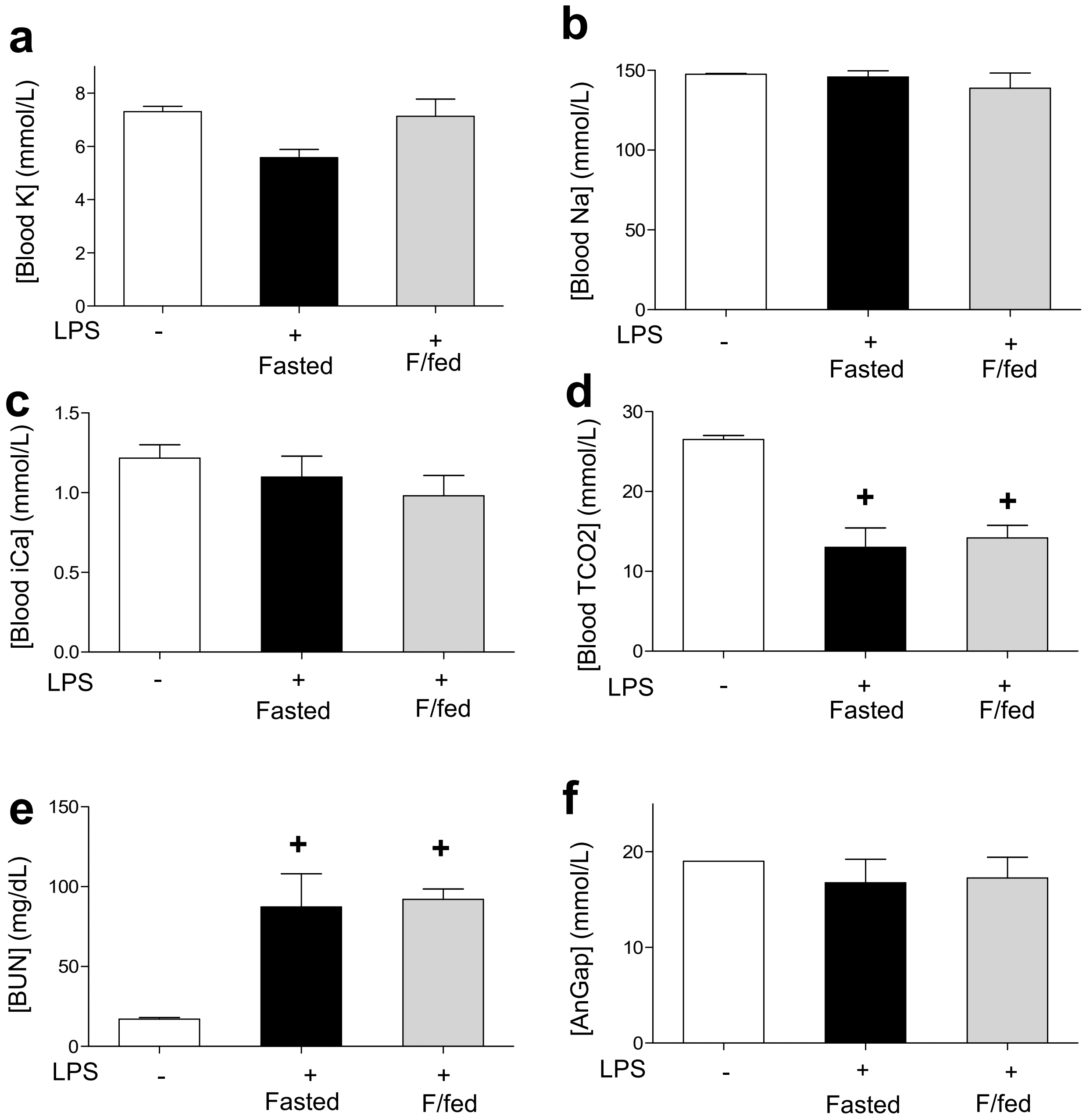

Supplementary Fig. 2. **Blood chemistry analyses of fasted and fed mice in endotoxemia.** Blood levels of **(a)** potassium(K),**(b)** sodium(Na),**(c)** ionized calcium(iCa),**(d)** total carbon dioxide(TCO2),**(e)** urea nitrogen(BUN), and **(f)** anion gap(AnGap) at 24h post-LPS. *p<0.05 vs LPS(n=4/group, One-way ANOVA).

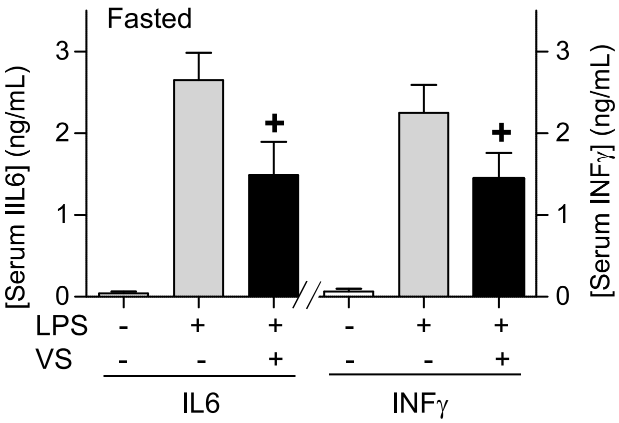

***Supplementary Fig.3.* Vagal stimulation attenuates serum levels of inflammatory cytokines in endotoxemia.** Fasted mice received vagal stimulation and the serum levels of IL-6 and INFg were analyzed at 4 hours post-LPS. +$p<0.05$ vs LPS(n=4/group; One-way ANOVA).
